# Continuous Intravenous Nimodipine Infusion With Ethanol as Carrier in Aneurysmal Subarachnoid Hemorrhage Does Not Result in Measurable Cerebral Ethanol Levels

**DOI:** 10.1002/cpt.3753

**Published:** 2025-06-24

**Authors:** Miriam M. Moser, Karl Rössler, Dorian Hirschmann, Leon Gramss, Walter Plöchl, Johannes Herta, Andrea Reinprecht, Markus Zeitlinger, Arthur Hosmann

**Affiliations:** ^1^ Department of Neurosurgery Medical University of Vienna Vienna Austria; ^2^ Department of Anesthesia, General Intensive Care Medicine and Pain Management Medical University of Vienna Vienna Austria; ^3^ Department of Clinical Pharmacology Medical University of Vienna Vienna Austria

## Abstract

An unimpaired neurological evaluation is essential for detecting delayed cerebral ischemia (DCI) in aneurysmal subarachnoid hemorrhage (aSAH) patients. Nimodipine is currently the only drug approved for DCI prevention. Intravenous nimodipine infusion contains 23.7 vol% ethanol as an excipient, resulting in up to 45 g of ethanol being infused daily, which may interfere with neurological assessment. Therefore, we aimed to measure ethanol concentrations in plasma, cerebrospinal fluid (CSF), and brain parenchyma during the infusion of 0.5–2 mg of nimodipine per hour in aSAH patients to quantify the ethanol neuronal exposure. Ethanol concentrations were determined by headspace gas chromatography‐flame ionization detection in plasma and CSF and we set out brain parenchyma measurement using cerebral microdialysis, in 10 aSAH patients. In each compartment four samples were taken over a 6‐hour interval during steady‐state intravenous nimodipine infusion at four different doses (0.5, 1, 1.5 and 2 mg/hour). A total of 307 samples from plasma and CSF was measured. Ethanol levels were mostly below the quantification limit of 0.002 g/100 mL. In 36 samples ethanol concentration was ≥ 0.002 g/100 mL, ranging from 0.002 to 0.009 g/100 mL. These low values were not reproducible in a second measurement, suggesting these values likely reflected analytical variability rather than true ethanol concentrations. Nimodipine analysis in brain parenchyma was omitted due to insufficient microdialysate volume and low concentrations in blood and CSF. Continuous nimodipine infusion of up to 2 mg/hour is unlikely to impair neurological assessment in aSAH patients, as no significant CSF ethanol concentration (< 0.002 g/100 mL) was detected.

**Study Highlights**

**WHAT IS THE CURRENT KNOWLEDGE ON THE TOPIC?**

Nimodipine is the only pharmacological agent approved for the prevention of ischemic neurological deficits following aSAH. The intravenous nimodipine formulation contains as an excipient 23.7 vol% ethanol, resulting in a daily ethanol exposure of up to 45 g at the maximum recommended infusion rate of 2 mg/hour. Despite its widespread use, little is known about the extent of ethanol penetration into the CSF and its potential neurotoxic effects in aSAH patients.

**WHAT QUESTION DID THIS STUDY ADDRESS?**

This study aimed to determine ethanol levels in the plasma and CSF to assess potential neuronal exposure to alcohol dependent on blood levels.

**WHAT DOES THIS STUDY ADD TO OUR KNOWLEDGE?**

So far, little is known about the extent of ethanol penetration into the CSF and its potential neurotoxic effects in aSAH patients.

**HOW MIGHT THIS CHANGE CLINICAL PHARMACOLOGY OR TRANSLATIONAL SCIENCE?**

The findings of this study assume that the ethanol content of the solution does not exert any toxic effect. On the contrary, earlier studies suggested that minimal ethanol doses may even have a beneficial effect. In this regard, intravenous nimodipine appears to be a safe treatment for patients following aSAH.

Aneurysmal subarachnoid hemorrhage (aSAH) results from the rupture of an intracranial aneurysm and is known to be associated with poor functional outcome, thereby causing a significant global public health risk.^1^, ^2^, ^3^ The initial hemorrhagic event is followed by secondary pathophysiological processes, including cerebral vasospasm and delayed cerebral ischemia, which further contribute to poor functional outcome.^4^, ^5^, ^6^, ^7^, ^8^

Nimodipine, a calcium channel blocker with high affinity to L‐type voltage‐gated calcium channels and strong specificity for cerebral arteries,^9^, ^10^, ^11^ is the only pharmacological agent approved for the prevention of ischemic neurological deficits following aSAH.^1^ Current guidelines recommend enteral nimodipine as a class Ia treatment (60 mg 6 times a day),^1^ and our group has recently shown that it is generally well tolerated from a hemodynamic perspective.^12^ While data remain limited,^1^, ^13^, ^14^ continuous intravenous infusion of nimodipine may be beneficial in critically ill patients by maintaining stable drug levels, avoiding peaks in plasma concentration, and ensuring more consistent cerebral blood flow.^14^, ^15^ Recently, we have also shown that intravenous administration achieves higher concentrations in both plasma and cerebrospinal fluid (CSF), which may translate into greater clinical effectiveness.^14^

However, the intravenous nimodipine formulation contains as an excipient 23.7 vol% ethanol, leading to a daily ethanol exposure of up to 45 g at the maximum recommended infusion rate of 2 mg/hour. Despite its widespread use, little is known about the extent of ethanol penetration into the CSF and its potential neurotoxic effects in aSAH patients.

Therefore, the objective of this study was to determine ethanol levels in the plasma, CSF, and brain parenchyma to enhance our pathophysiological understanding of its toxic effects to neurons and quantify the neuronal exposure to alcohol dependent on blood levels. To our knowledge, this fundamental research question has not been previously investigated.

## MATERIALS AND METHODS

### Population

Ten patients were included in this prospective study between 11/2020 and 10/2022. All of them suffered from severe aSAH requiring deep sedation, multimodal neuromonitoring, and cerebral microdialysis.

The excipient ethanol has been studied during continuous infusion at steady state of the study drug nimodipine.

The ethics committee of the Medical University of Vienna (EK‐Nr. 1774/2020, EudraCT 2020‐002968‐31) approved the study protocol, and the study was conducted at the neurosurgical intensive care unit of the Medical University of Vienna. All study procedures adhered to the principles of the Declaration of Helsinki of 1975. The patients who met the study criteria were initially unable to provide written consent due to deep sedation and mechanical ventilation. Patients were informed about the study when they regained consciousness.

### Nimodipine

**Figure 1:**
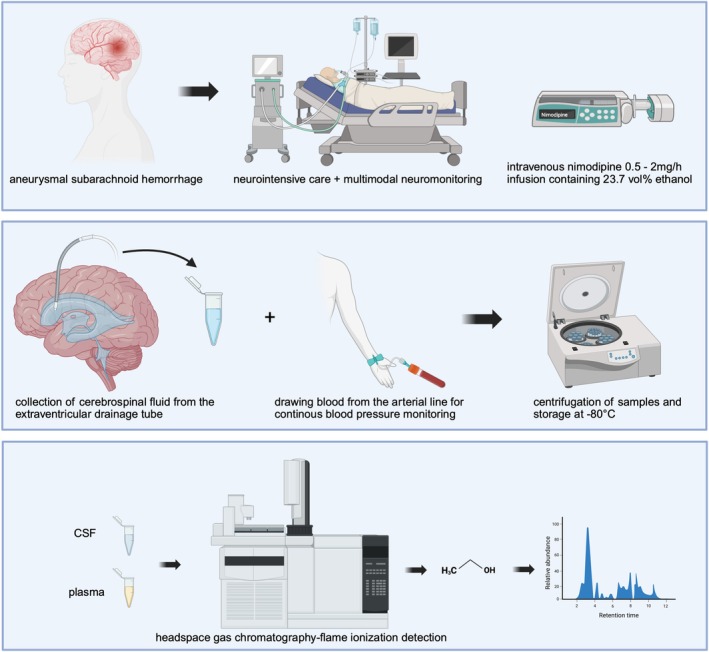
Study set‐up. Created in https://BioRender.com.

Nimodipine was continuously administered intravenously to all included patients with aSAH to prevent delayed cerebral ischemia. Ethanol concentrations were measured at steady state of four different nimodipine infusion rates (0.5, 1.0, 1.5, and 2.0 mg/hour). The infusion solution contains 23.7 vol% ethanol.^16^, ^17^ This results in the infusion of 11.21, 22.42, 33.63, and 44.87 g of ethanol over 24 hours at infusion rates of 0.5, 1.0, 1.5, and 2.0 mg/hour, respectively. For each infusion rate, measurements of ethanol were performed at four different timepoints (i.e., after 1, 3, 5 and 7 hours on the respective study day). Elimination half‐life of intravenous administration of nimodipine is reported to range between 0.9 and 1.5 hours.^18^ To ensure that steady‐state levels had been reached, nimodipine dose was changed at least 12 hours prior to pharmacokinetic (PK) measurement (**Figure** **1**). Samples were centrifuged and stored immediately at −80°C to minimize evaporation, chemical oxidation, or microbial consumption.

### Sampling

Ethanol concentrations in plasma and CSF were measured at steady state doses of 0.5, 1, 1.5, and 2 mg/hour nimodipine. Four samples were collected in plasma and CSF at each dose, with a 2‐hour interval between the samples. Five milliliters of blood were obtained from an arterial catheter for plasma measurement. CSF was collected from an external ventricular drainage. The first milliliter of CSF was discarded, and the subsequent milliliter was retained and stored for further analysis. All samples were kept on ice, immediately centrifuged, and stored at −80°C thereafter (**Figure** **1**).

A Bolt Microdialysis Catheter (M Dialysis AB, Stockholm, Sweden) was inserted in the frontal lobe, side‐by‐side with a NEUROVENT‐PTO 2L catheter (Raumedic AG, Helmbrechts, Germany), on the side of the ruptured aneurysm or where the extension of the subarachnoid blood was at its maximum. The microdialysis catheter was continuously perfused with artificial CSF (Perfusion Fluid CNS, M Dialysis AB, Stockholm, Sweden) at a flow rate of 0.3 μL/minute, using a 107 Microdialysis Pump (M Dialysis AB, Stockholm, Sweden). Samples for analysis were collected every hour, alternately once for routine bedside analysis of the cerebral metabolism and every other hour for research purposes. Due to the limited volume of microdialysate and the very low concentrations observed in blood and CSF, the focus of the analysis was ultimately directed toward these two compartments.

### Drug assay

Ethanol was quantified in serum and CSF by headspace gas chromatography‐flame ionization detection using a Clarus® 580 gas chromatograph hyphenated with a TurboMatrix Headspace sampler (both PerkinElmer, Waltham, USA). Each sample was analyzed twice for the reproducibility of the results. Chromatographic separation was carried out using two columns with different separation properties (Elite‐BAC‐1 Advantage 30 m × 0.32 mm × 1.80 μm, Elite‐BAC‐2 Advantage 30 m × 0.32 mm × 0.60 μm, both PerkinElmer). Sample preparation was limited to the dilution of 100 μL sample with 1.0 mL water containing 0.1 g/L tert‐butanol as internal standard. Ethanol‐negative and ethanol‐positive samples at different concentration levels served as quality control samples. The detection and quantification limit was set at 0.02 g/L (≙ 0.002 g/100 mL), corresponding to a blood alcohol content (BAC) of 0.002%. Samples were analyzed by the “FTC – Forensisch Toxikologisches Labor” in Vienna, Austria (**Figure** **1**).

## RESULTS

### Population

This prospective study included 10 patients with severe aSAH treated between 11/2020 and 10/2022. The patients were admitted with a mean Hunt & Hess grade of 4 ± 1 that required deep sedation. Patients' demographics are presented in detail in **Table** **1**. During the observation period, there was a slight increase in liver function parameters (**Table** **1**).

**Table 1:** Patients' characteristics

Patients included	10
Age (years)	55 ± 10 (range 36–68)
Sex	
Female	6
Male	4
Hunt & Hess	4 ± 1
Body weight (kg)	70 ± 12
Average BMI	23 ± 2
Surgical intervention	
Clipping	5
Coiling	5
Ethanol analysis after aSAH (days)	
0.5 mg/h i.v.	5 (±3)
1 mg/h i.v.	6 (±2)
1.5 mg/h i.v.	8 (±5)
2 mg/h i.v.	9 (±2)
Liver parameters	[mean ± SD]
Alkaline phosphatase	
0.5 mg/hour	94 ± 49 U/L
1 mg/hour	105 ± 35 U/L
1.5 mg/hour	226 ± 307 U/L
2 mg/hour	282 ± 249 U/L
Gamma‐GT	
0.5 mg/hour	116 ± 103 U/L
1 mg/hour	127 ± 93 U/L
1.5 mg/hour	246 ± 262 U/L
2 mg/hour	314 ± 182 U/L
GOT	
0.5 mg/hour	35 ± 19 U/L
1 mg/hour	44 ± 19 U/L
1.5 mg/hour	57 ± 48 U/L
2 mg/hour	69 ± 46 U/L
GPT	
0.5 mg/hour	37 ± 30 U/L
1 mg/hour	43 ± 23 U/L
1.5 mg/hour	62 ± 51 U/L
2 mg/hour	74 ± 45 U/L
Total bilirubin	
0.5 mg/hour	0.5 ± 0.57 mg/dL
1 mg/hour	0.61 ± 0.64 mg/dL
1.5 mg/hour	1.13 ± 2.12 mg/dL
2 mg/hour	0.71 ± 1.2 mg/dL

Data are presented as mean ± standard deviation. Local laboratory references: alkaline phosphatase [35–105 U/L]; gamma‐GT [< 40 U/L]; GOT [< 35 U/L]; GPT [< 35 U/L]; total bilirubin [0.0–1.2 mg/dL]. aSAH, aneurysmal subarachnoid hemorrhage; BMI, body mass index.

Ethanol concentrations were measured in plasma and CSF concomitantly at different steady‐state doses of nimodipine between 5 ± 3 and 9 ± 2 days after aSAH (**Table** **1**).

Four blood samples were collected for each nimodipine dose at steady state, except for one patient, where one sample is missing at 0.5 mg/hour and one at 1 mg/hour nimodipine infusion, resulting in a total of 158 plasma samples. In one patient, CSF could not be obtained at a dose of 0.5 mg/hour due to slit ventricles. In four other patients, temporary slit ventricles prevented sample collection at one or, at most, two of the four time points per dose. As a result, a total of 149 CSF samples were collected. Analysis of ethanol in brain parenchyma was omitted due to the limited volume of microdialysate and the very low concentrations in both blood and CSF.

### Plasma and CSF ethanol concentration

We analyzed in total 307 plasma and CSF samples in 10 patients after aSAH at four intravenous nimodipine doses (0.5, 1, 1.5, and 2 mg/hour). Ethanol levels were generally below the detection limit of 0.002 g/100 mL. In 36 samples, the ethanol concentration in one of the two measurements performed for each sample was ≥ 0.002 g/100 mL (**Table** **2**).

**Table 2:** Ethanol concentration in plasma and cerebrospinal fluid measured using headspace gas chromatography‐flame ionization detection

Patient	Time point on study day (hours)	Compartment	Dose of nimodipine (mg/hour)	Measured concentration 1 (g/100 mL)	Measured concentration 2 (g/100 mL)
04	5	CSF	0.5	< 0.002	0.0022
04	7	CSF	0.5	< 0.002	0.00297
04	1	CSF	1	< 0.002	0.00241
04	1	CSF	1.5	< 0.002	0.00494
04	3	CSF	1.5	< 0.002	0.00366
04	5	CSF	1.5	< 0.002	0.00347
04	7	CSF	1.5	< 0.002	0.00304
07	1	CSF	1	< 0.002	0.00208
07	3	Plasma	1.5	< 0.002	0.00236
07	5	Plasma	1.5	< 0.002	0.00322
07	7	Plasma	1.5	< 0.002	0.00353
07	1	Plasma	2	< 0.002	0.00431
07	3	Plasma	2	< 0.002	0.00416
07	5	Plasma	2	< 0.002	0.00445
07	7	Plasma	2	< 0.002	0.00432
08	1	CSF	0.5	< 0.002	0.002
08	3	CSF	0.5	< 0.002	0.00234
08	5	CSF	0.5	< 0.002	0.00267
08	7	CSF	0.5	< 0.002	0.00322
08	1	CSF	1	< 0.002	0.00373
08	3	CSF	1	< 0.002	0.00406
08	5	CSF	1	< 0.002	0.00442
08	7	CSF	1	< 0.002	0.00866
08	5	CSF	1.5	< 0.002	0.00208
08	1	Plasma	1.5	< 0.002	0.00923
08	3	Plasma	1.5	< 0.002	0.0082
08	5	Plasma	1.5	< 0.002	0.00776
08	7	Plasma	1.5	< 0.002	0.00775
08	1	Plasma	2	< 0.002	0.00226
08	3	Plasma	2	< 0.002	0.0027
08	5	Plasma	2	< 0.002	0.00242
08	7	Plasma	2	< 0.002	0.00221
09	1	Plasma	2	< 0.002	0.00261
09	3	Plasma	2	< 0.002	0.00281
09	5	Plasma	2	< 0.002	0.00258
09	7	Plasma	2	< 0.002	0.00237

See **Table** **S1** for details on each measured sample. CSF, cerebrospinal fluid; h, hours.

In one of two measurements we measured values ranging from 0.002 to 0.009 g/100 mL, but those were not reproducible in the second measurement, suggesting they are likely due to analytical variability rather than true quantifiable ethanol concentrations (**Table** **2**).

## DISCUSSION

In this prospective study, we analyzed ethanol concentrations in plasma and CSF for the first time, to the best of our knowledge, in patients receiving continuous intravenous infusion of nimodipine after severe aSAH. We measured blood ethanol levels and ethanol concentrations in CSF in humans and found no detectable ethanol in these compartments.

aSAH still results in bad long‐term neurological outcome due to delayed cerebral ischemia.^1^, ^4^ Possible advantages of intravenous nimodipine infusion over oral administration include continuous drug exposure in plasma and, consequently, also in CSF, ensuring sustained brain exposure to nimodipine and its putative neuroprotective effects.^19^, ^20^ In addition, gastrointestinal dysfunction is an increasingly recognized problem in intensive care patients,^21^ which may affect drug distribution after oral intake. However, this has not yet been specifically studied in neurointensive care patients, leaving a gap in the current evidence in this regard.^1^

The intravenous formulation of nimodipine contains ethanol as a carrier solution,^17^ due to the poor solubility of nimodipine in alternative carrier solutions such as 0.9% NaCl or 5% glucose.^16^, ^17^, ^22^ Although ethanol intakes of up to 45 g/day could theoretically impair neurological assessment, pharmacokinetic estimations suggest that a continuous infusion of 2 mg/hour nimodipine introduces approximately 1.87 g ethanol per hour, resulting in a theoretical maximum blood alcohol concentration (BAC) of ~0.0045% when distributed across total body water (≈ 42 L in a 70 kg adult). This concentration lies near the analytical detection limit (0.002%) and is well below the typical hepatic elimination rate (~0.015% per hour), making clinically relevant effects highly unlikely. Notably, the present study substantiates these estimations by demonstrating that systemic ethanol concentrations during infusion remain negligible, thus confirming that the ethanol content in the intravenous nimodipine formulation does not reach levels associated with neurotoxicity or impairment of neurological evaluation.

Laboratory findings indicated a mild elevation of liver function parameters during the course of nimodipine administration. However, this increase is more likely attributable to factors such as the underlying critical illness, concomitant antibiotic therapy^23^ and possibly prolonged sedation with esketamine,^24^ rather than the ethanol content of intravenous nimodipine solution. Notably, the elevation in liver enzymes appeared to follow a time‐dependent pattern rather than a dose‐dependent one. This interpretation is supported by the observation that nimodipine dosing was not consistently titrated in an ascending manner—lower doses (e.g., 0.5 mg/hour, 1.5 mg/hour) were in some instances administered later than higher doses (e.g., 2 mg/hour). The absence of a direct relationship between dose and enzyme elevation further suggests that the observed changes in liver function were not related to the amount of ethanol infused but rather to the clinical course of critically ill patients receiving intensive care.^23^, ^24^, ^25^

Interestingly, the ethanol carrier solution itself may contribute to cerebral neuroprotection and reduced hypoxia following aSAH.^22^ Early mouse studies^22^ compared the effects of ethanol alone, nimodipine diluted in saline, and nimodipine diluted in ethanol on hypoxia, showing a significant positive effect of ethanol in reducing hypoxia. This led us to the conclusion that ethanol, as a carrier solution, may be an overlooked contributor to the beneficial effects of nimodipine on outcome after aSAH. However, a direct comparison between nimodipine diluted in saline and in ethanol is challenging, given the poor solubility of nimodipine in saline.^16^ It is possible that nimodipine's full therapeutic potential has not been achieved in the saline‐based solution. However, since ethanol alone also showed neuroprotective effects, these findings support the conclusion that the carrier solution itself plays a role in the drug's overall effect.

In our study, no measurable ethanol concentrations were detected at a quantification limit of 0.002 g/100 mL, equivalent to 0.002% BAC. Nonetheless, it remains possible that negligible concentrations, which are continuously degraded by the liver, kidney, lungs, and skin,^26^, ^27^ do not induce toxic effects on brain tissue while still providing a protective effect against hypoxia in addition to the beneficial effects of nimodipine. These findings suggest that the observed effects may not be attributable exclusively to nimodipine itself. Future in vitro studies and clinical outcome comparisons would be of interest to further explore this phenomenon.

Importantly, the effects of ethanol on the human brain vary depending on the dose administered and its metabolites. While moderate doses showed a reduced stroke risk, higher doses lead to increased cerebral arterial contraction.^28^ A rodent study showed significantly higher ethanol levels in the CSF compared to blood after oral administration of 3 g ethanol/kg.^29^ Peak values were reached within the CSF at 44.8 ± 10.9 μmol/mL after 60 minutes and within the blood at 27.7 ± 6.0 μmol/mL after 120 minutes.^29^ When analyzing alcohol levels in neurocritical care patients, endogenous ethanol production must be considered under pathological conditions. In one study, higher ethanol levels were observed in CSF (43.7 ± 28.9 mg%) than in blood (38.5 ± 35.7 mg%) under pathological conditions, that is, neurocysticercosis, brain aneurysm, epilepsy, cranioencephalic trauma, guillain‐barré polyradiculoneuritis, bacterial meningitis, central nervous system neoplasia, multiple sclerosis, and others. Patients with pathological CSF revealed even higher ethanol levels than those with physiological CSF readings.^30^

A post‐mortem study of intoxicated cadavers comparing ethanol concentrations in femoral blood and CSF during forensic autopsy revealed a strong correlation of blood and CSF levels (*R* ^2^ = 96%), with CSF levels approximately 20–30% higher.^31^ The higher CSF concentration of ethanol is most likely attributed to the higher water content of CSF.^31^ Although the exact amount of alcohol consumed by those individuals cannot be precisely reconstructed, it is reasonable to assume that they were highly alcohol intoxicated. Given that one per mille roughly corresponds to 1 mg/kg of blood alcohol concentration,^32^ it can be concluded that even at the highest nimodipine infusion rate of 2 mg/hour, the resulting blood alcohol concentration would be far below the level observed in these cadavers. However, the precise threshold at which ethanol might accumulate in CSF to a significant extent remains to be determined.

In our patient population, continuous nimodipine administration of 2 mg/hour resulted in an ethanol intake of up to 45 g/day, corresponding to less than 1 g ethanol/kg. Ethanol levels remained below the detection limit of 0.002 g/100 mL of ethanol, equivalent to 0.002% BAC.

We could show that ethanol is below the limit of detection in the human brain at these low dose infusions and, therefore, does not contribute to further neurological damage. On the contrary, trace amounts below the detection limit may even exert a protective effect. However, this hypothesis needs to be explored in future studies.

## LIMITATIONS

The number of patients included in this study was limited due to the challenges associated with obtaining samples from plasma, CSF, and brain parenchyma, which restricted the diversity of our dataset. Additionally, our measurement sensitivity was constrained to a lower detection limit of 0.02 g/100 mL, preventing the identification of even smaller concentrations and their potential effects. Ethanol‐positive and ethanol‐negative samples served as quality control; however, this is not a placebo‐controlled study, thus no direct positive control samples from patients' blood or CSF were available. Furthermore, the analysis was restricted to pharmacokinetic assessment and did not include pharmacodynamic evaluation.

## CONCLUSION

No significant ethanol concentrations were detected in plasma and CSF, despite the carrier solution of nimodipine containing 23.7 vol% ethanol, leading to a daily ethanol exposure of up to 45 g at the maximum recommended infusion rate of 2 mg/hour. This indicates that the ethanol content of the solution does not exert any toxic effect. On the contrary, studies suggest that minimal ethanol doses may even have a beneficial effect. In this regard, intravenous nimodipine appears to be a safe treatment for patients following aSAH. However, further prospective studies are needed to determine whether intravenous administration leads to superior clinical outcomes compared to the current standard of care with oral nimodipine.

## FUNDING

This study was funded by the Austrian Science Fund (FWF): (KLI 947‐B).

## CONFLICT OF INTEREST

The authors declared no competing interests for this work.

## AUTHOR CONTRIBUTIONS

K.R., A.H., M.M.M., and M.Z. designed the research, M.M.M. and A.H. wrote the manuscript, M.M.M., K.R., D.H., L.G., W.P., J.H., A.R., and M.Z. performed the research, M.M.M. and A.H. analyzed the data.

## Supporting information


Table S1

## Data Availability

The datasets used and/or analyzed during the current study are available from the corresponding author on reasonable request.
