# Supplementary material for: Continuous Intravenous Nimodipine Infusion With Ethanol as Carrier in Aneurysmal Subarachnoid Hemorrhage Does Not Result in Measurable Cerebral Ethanol Levels
Source: Clin Pharmacol Ther. 2025 Jun 24;118(4):928–34. doi: 10.1002/cpt.3753 (PMC12439008; doi:10.1002/cpt.3753)
Supplement: Supplementary file 1 — Table S1 [file CPT-118-928-s001.docx]

*Table S1: Ethanol concentration in plasma and cerebrospinal fluid in detail using headspace gas chromatography-flame ionization detection. CSF = cerebrospinal fluid, h = hour.*

| Patient | Time point on study day | Compartment | Dose of Nimodipine | Measured concentration 1 [g/100mL] | Measured concentration 2 [g/100mL] |
| --- | --- | --- | --- | --- | --- |
| 01 | 1h | CSF | 0.5 mg/h | <0.002 | <0.002 |
| 01 | 3h | CSF | 0.5 mg/h | <0.002 | <0.002 |
| 01 | 5h | CSF | 0.5 mg/h | <0.002 | <0.002 |
| 01 | 7h | CSF | 0.5 mg/h | <0.002 | <0.002 |
| 01 | 1h | CSF | 1 mg/h | <0.002 | <0.002 |
| 01 | 3h | CSF | 1 mg/h | <0.002 | <0.002 |
| 01 | 5h | CSF | 1 mg/h | <0.002 | <0.002 |
| 01 | 7h | CSF | 1 mg/h | <0.002 | <0.002 |
| 01 | 1h | CSF | 1.5 mg/h | <0.002 | <0.002 |
| 01 | 3h | CSF | 1.5 mg/h | <0.002 | <0.002 |
| 01 | 5h | CSF | 1.5 mg/h | <0.002 | <0.002 |
| 01 | 7h | CSF | 1.5 mg/h | <0.002 | <0.002 |
| 01 | 1h | CSF | 2 mg/h | <0.002 | <0.002 |
| 01 | 3h | CSF | 2 mg/h | <0.002 | <0.002 |
| 01 | 5h | CSF | 2 mg/h | <0.002 | <0.002 |
| 01 | 7h | CSF | 2 mg/h | <0.002 | <0.002 |
| 01 | 1h | Plasma | 0.5 mg/h | <0.002 | <0.002 |
| 01 | 3h | Plasma | 0.5 mg/h | <0.002 | <0.002 |
| 01 | 5h | Plasma | 0.5 mg/h | <0.002 | <0.002 |
| 01 | 7h | Plasma | 0.5 mg/h | <0.002 | <0.002 |
| 01 | 1h | Plasma | 1 mg/h | <0.002 | <0.002 |
| 01 | 3h | Plasma | 1 mg/h | <0.002 | <0.002 |
| 01 | 5h | Plasma | 1 mg/h | <0.002 | <0.002 |
| 01 | 7h | Plasma | 1 mg/h | <0.002 | <0.002 |
| 01 | 1h | Plasma | 1.5 mg/h | <0.002 | <0.002 |
| 01 | 3h | Plasma | 1.5 mg/h | <0.002 | <0.002 |
| 01 | 5h | Plasma | 1.5 mg/h | <0.002 | <0.002 |
| 01 | 7h | Plasma | 1.5 mg/h | <0.002 | <0.002 |
| 01 | 1h | Plasma | 2 mg/h | <0.002 | <0.002 |
| 01 | 3h | Plasma | 2 mg/h | <0.002 | <0.002 |
| 01 | 5h | Plasma | 2 mg/h | <0.002 | <0.002 |
| 01 | 7h | Plasma | 2 mg/h | <0.002 | <0.002 |
| 02 | 1h | CSF | 0.5 mg/h | <0.002 | <0.002 |
| 02 | 5h | CSF | 0.5 mg/h | <0.002 | <0.002 |
| 02 | 1h | CSF | 1 mg/h | <0.002 | <0.002 |
| 02 | 3h | CSF | 1 mg/h | <0.002 | <0.002 |
| 02 | 5h | CSF | 1 mg/h | <0.002 | <0.002 |
| 02 | 7h | CSF | 1 mg/h | <0.002 | <0.002 |
| 02 | 1h | CSF | 1.5 mg/h | <0.002 | <0.002 |
| 02 | 3h | CSF | 1.5 mg/h | <0.002 | <0.002 |
| 02 | 5h | CSF | 1.5 mg/h | <0.002 | <0.002 |
| 02 | 1h | CSF | 2 mg/h | <0.002 | <0.002 |
| 02 | 3h | CSF | 2 mg/h | <0.002 | <0.002 |
| 02 | 7h | CSF | 2 mg/h | <0.002 | <0.002 |
| 02 | 1h | Plasma | 0.5 mg/h | <0.002 | <0.002 |
| 02 | 3h | Plasma | 0.5 mg/h | <0.002 | <0.002 |
| 02 | 5h | Plasma | 0.5 mg/h | <0.002 | <0.002 |
| 02 | 7h | Plasma | 0.5 mg/h | <0.002 | <0.002 |
| 02 | 1h | Plasma | 1 mg/h | <0.002 | <0.002 |
| 02 | 3h | Plasma | 1 mg/h | <0.002 | <0.002 |
| 02 | 5h | Plasma | 1 mg/h | <0.002 | <0.002 |
| 02 | 7h | Plasma | 1 mg/h | <0.002 | <0.002 |
| 02 | 1h | Plasma | 1.5 mg/h | <0.002 | <0.002 |
| 02 | 3h | Plasma | 1.5 mg/h | <0.002 | <0.002 |
| 02 | 5h | Plasma | 1.5 mg/h | <0.002 | <0.002 |
| 02 | 7h | Plasma | 1.5 mg/h | <0.002 | <0.002 |
| 02 | 1h | Plasma | 2 mg/h | <0.002 | <0.002 |
| 02 | 3h | Plasma | 2 mg/h | <0.002 | <0.002 |
| 02 | 5h | Plasma | 2 mg/h | <0.002 | <0.002 |
| 02 | 7h | Plasma | 2 mg/h | <0.002 | <0.002 |
| 03 | 1h | CSF | 0.5 mg/h | <0.002 | <0.002 |
| 03 | 3h | CSF | 0.5 mg/h | <0.002 | <0.002 |
| 03 | 5h | CSF | 0.5 mg/h | <0.002 | <0.002 |
| 03 | 7h | CSF | 0.5 mg/h | <0.002 | <0.002 |
| 03 | 1h | CSF | 1 mg/h | <0.002 | <0.002 |
| 03 | 3h | CSF | 1 mg/h | <0.002 | <0.002 |
| 03 | 5h | CSF | 1 mg/h | <0.002 | <0.002 |
| 03 | 7h | CSF | 1 mg/h | <0.002 | <0.002 |
| 03 | 1h | CSF | 1.5 mg/h | <0.002 | <0.002 |
| 03 | 3h | CSF | 1.5 mg/h | <0.002 | <0.002 |
| 03 | 5h | CSF | 1.5 mg/h | <0.002 | <0.002 |
| 03 | 7h | CSF | 1.5 mg/h | <0.002 | <0.002 |
| 03 | 1h | CSF | 2 mg/h | <0.002 | <0.002 |
| 03 | 3h | CSF | 2 mg/h | <0.002 | <0.002 |
| 03 | 5h | CSF | 2 mg/h | <0.002 | <0.002 |
| 03 | 7h | CSF | 2 mg/h | <0.002 | <0.002 |
| 03 | 1h | Plasma | 0.5 mg/h | <0.002 | <0.002 |
| 03 | 3h | Plasma | 0.5 mg/h | <0.002 | <0.002 |
| 03 | 5h | Plasma | 0.5 mg/h | <0.002 | <0.002 |
| 03 | 7h | Plasma | 0.5 mg/h | <0.002 | <0.002 |
| 03 | 1h | Plasma | 1 mg/h | <0.002 | <0.002 |
| 03 | 3h | Plasma | 1 mg/h | <0.002 | <0.002 |
| 03 | 5h | Plasma | 1 mg/h | <0.002 | <0.002 |
| 03 | 7h | Plasma | 1 mg/h | <0.002 | <0.002 |
| 03 | 1h | Plasma | 1.5 mg/h | <0.002 | <0.002 |
| 03 | 3h | Plasma | 1.5 mg/h | <0.002 | <0.002 |
| 03 | 5h | Plasma | 1.5 mg/h | <0.002 | <0.002 |
| 03 | 7h | Plasma | 1.5 mg/h | <0.002 | <0.002 |
| 03 | 1h | Plasma | 2 mg/h | <0.002 | <0.002 |
| 03 | 3h | Plasma | 2 mg/h | <0.002 | <0.002 |
| 03 | 5h | Plasma | 2 mg/h | <0.002 | <0.002 |
| 03 | 7h | Plasma | 2 mg/h | <0.002 | <0.002 |
| 04 | 1h | CSF | 0.5 mg/h | <0.002 | <0.002 |
| 04 | 3h | CSF | 0.5 mg/h | <0.002 | <0.002 |
| 04 | 5h | CSF | 0.5 mg/h | <0.002 | 0.0022 |
| 04 | 7h | CSF | 0.5 mg/h | <0.002 | 0.00297 |
| 04 | 1h | CSF | 1 mg/h | <0.002 | 0.00241 |
| 04 | 3h | CSF | 1 mg/h | <0.002 | <0.002 |
| 04 | 5h | CSF | 1 mg/h | <0.002 | <0.002 |
| 04 | 7h | CSF | 1 mg/h | <0.002 | <0.002 |
| 04 | 1h | CSF | 1.5 mg/h | <0.002 | 0.00494 |
| 04 | 3h | CSF | 1.5 mg/h | <0.002 | 0.00366 |
| 04 | 5h | CSF | 1.5 mg/h | <0.002 | 0.00347 |
| 04 | 7h | CSF | 1.5 mg/h | <0.002 | 0.00304 |
| 04 | 3h | CSF | 2 mg/h | <0.002 | <0.002 |
| 04 | 5h | CSF | 2 mg/h | <0.002 | <0.002 |
| 04 | 1h | Plasma | 0.5 mg/h | <0.002 | <0.002 |
| 04 | 3h | Plasma | 0.5 mg/h | <0.002 | <0.002 |
| 04 | 5h | Plasma | 0.5 mg/h | <0.002 | <0.002 |
| 04 | 7h | Plasma | 0.5 mg/h | <0.002 | <0.002 |
| 04 | 1h | Plasma | 1 mg/h | <0.002 | <0.002 |
| 04 | 3h | Plasma | 1 mg/h | <0.002 | <0.002 |
| 04 | 5h | Plasma | 1 mg/h | <0.002 | <0.002 |
| 04 | 1h | Plasma | 1.5 mg/h | <0.002 | <0.002 |
| 04 | 3h | Plasma | 1.5 mg/h | <0.002 | <0.002 |
| 04 | 5h | Plasma | 1.5 mg/h | <0.002 | <0.002 |
| 04 | 7h | Plasma | 1.5 mg/h | <0.002 | <0.002 |
| 04 | 1h | Plasma | 2 mg/h | <0.002 | <0.002 |
| 04 | 3h | Plasma | 2 mg/h | <0.002 | <0.002 |
| 04 | 5h | Plasma | 2 mg/h | <0.002 | <0.002 |
| 04 | 7h | Plasma | 2 mg/h | <0.002 | <0.002 |
| 05 | 1h | CSF | 0.5 mg/h | <0.002 | <0.002 |
| 05 | 3h | CSF | 0.5 mg/h | <0.002 | <0.002 |
| 05 | 5h | CSF | 0.5 mg/h | <0.002 | <0.002 |
| 05 | 7h | CSF | 0.5 mg/h | <0.002 | <0.002 |
| 05 | 1h | CSF | 1 mg/h | <0.002 | <0.002 |
| 05 | 3h | CSF | 1 mg/h | <0.002 | <0.002 |
| 05 | 5h | CSF | 1 mg/h | <0.002 | <0.002 |
| 05 | 7h | CSF | 1 mg/h | <0.002 | <0.002 |
| 05 | 1h | CSF | 1.5 mg/h | <0.002 | <0.002 |
| 05 | 3h | CSF | 1.5 mg/h | <0.002 | <0.002 |
| 05 | 5h | CSF | 1.5 mg/h | <0.002 | <0.002 |
| 05 | 7h | CSF | 1.5 mg/h | <0.002 | <0.002 |
| 05 | 1h | CSF | 2 mg/h | <0.002 | <0.002 |
| 05 | 3h | CSF | 2 mg/h | <0.002 | <0.002 |
| 05 | 5h | CSF | 2 mg/h | <0.002 | <0.002 |
| 05 | 7h | CSF | 2 mg/h | <0.002 | <0.002 |
| 05 | 1h | Plasma | 0.5 mg/h | <0.002 | <0.002 |
| 05 | 3h | Plasma | 0.5 mg/h | <0.002 | <0.002 |
| 05 | 5h | Plasma | 0.5 mg/h | <0.002 | <0.002 |
| 05 | 7h | Plasma | 0.5 mg/h | <0.002 | <0.002 |
| 05 | 1h | Plasma | 1 mg/h | <0.002 | <0.002 |
| 05 | 3h | Plasma | 1 mg/h | <0.002 | <0.002 |
| 05 | 5h | Plasma | 1 mg/h | <0.002 | <0.002 |
| 05 | 7h | Plasma | 1 mg/h | <0.002 | <0.002 |
| 05 | 1h | Plasma | 1.5 mg/h | <0.002 | <0.002 |
| 05 | 3h | Plasma | 1.5 mg/h | <0.002 | <0.002 |
| 05 | 5h | Plasma | 1.5 mg/h | <0.002 | <0.002 |
| 05 | 7h | Plasma | 1.5 mg/h | <0.002 | <0.002 |
| 05 | 1h | Plasma | 2 mg/h | <0.002 | <0.002 |
| 05 | 3h | Plasma | 2 mg/h | <0.002 | <0.002 |
| 05 | 5h | Plasma | 2 mg/h | <0.002 | <0.002 |
| 05 | 7h | Plasma | 2 mg/h | <0.002 | <0.002 |
| 06 | 1h | CSF | 1 mg/h | <0.002 | <0.002 |
| 06 | 3h | CSF | 1 mg/h | <0.002 | <0.002 |
| 06 | 5h | CSF | 1 mg/h | <0.002 | <0.002 |
| 06 | 7h | CSF | 1 mg/h | <0.002 | <0.002 |
| 06 | 1h | CSF | 1.5 mg/h | <0.002 | <0.002 |
| 06 | 3h | CSF | 1.5 mg/h | <0.002 | <0.002 |
| 06 | 5h | CSF | 1.5 mg/h | <0.002 | <0.002 |
| 06 | 7h | CSF | 1.5 mg/h | <0.002 | <0.002 |
| 06 | 1h | CSF | 2 mg/h | <0.002 | <0.002 |
| 06 | 3h | CSF | 2 mg/h | <0.002 | <0.002 |
| 06 | 5h | CSF | 2 mg/h | <0.002 | <0.002 |
| 06 | 7h | CSF | 2 mg/h | <0.002 | <0.002 |
| 06 | 1h | Plasma | 0.5 mg/h | <0.002 | <0.002 |
| 06 | 3h | Plasma | 0.5 mg/h | <0.002 | <0.002 |
| 06 | 5h | Plasma | 0.5 mg/h | <0.002 | <0.002 |
| 06 | 7h | Plasma | 0.5 mg/h | <0.002 | <0.002 |
| 06 | 1h | Plasma | 1 mg/h | <0.002 | <0.002 |
| 06 | 3h | Plasma | 1 mg/h | <0.002 | <0.002 |
| 06 | 5h | Plasma | 1 mg/h | <0.002 | <0.002 |
| 06 | 7h | Plasma | 1 mg/h | <0.002 | <0.002 |
| 06 | 1h | Plasma | 1.5 mg/h | <0.002 | <0.002 |
| 06 | 3h | Plasma | 1.5 mg/h | <0.002 | <0.002 |
| 06 | 5h | Plasma | 1.5 mg/h | <0.002 | <0.002 |
| 06 | 7h | Plasma | 1.5 mg/h | <0.002 | <0.002 |
| 06 | 1h | Plasma | 2 mg/h | <0.002 | <0.002 |
| 06 | 3h | Plasma | 2 mg/h | <0.002 | <0.002 |
| 06 | 5h | Plasma | 2 mg/h | <0.002 | <0.002 |
| 06 | 7h | Plasma | 2 mg/h | <0.002 | <0.002 |
| 07 | 1h | CSF | 0.5 mg/h | <0.002 | <0.002 |
| 07 | 3h | CSF | 0.5 mg/h | <0.002 | <0.002 |
| 07 | 5h | CSF | 0.5 mg/h | <0.002 | <0.002 |
| 07 | 7h | CSF | 0.5 mg/h | <0.002 | <0.002 |
| 07 | 1h | CSF | 1 mg/h | <0.002 | 0.00208 |
| 07 | 3h | CSF | 1 mg/h | <0.002 | <0.002 |
| 07 | 5h | CSF | 1 mg/h | <0.002 | <0.002 |
| 07 | 7h | CSF | 1 mg/h | <0.002 | <0.002 |
| 07 | 1h | CSF | 1.5 mg/h | <0.002 | <0.002 |
| 07 | 3h | CSF | 1.5 mg/h | <0.002 | <0.002 |
| 07 | 5h | CSF | 1.5 mg/h | <0.002 | <0.002 |
| 07 | 7h | CSF | 1.5 mg/h | <0.002 | <0.002 |
| 07 | 1h | CSF | 2 mg/h | <0.002 | <0.002 |
| 07 | 3h | CSF | 2 mg/h | <0.002 | <0.002 |
| 07 | 5h | CSF | 2 mg/h | <0.002 | <0.002 |
| 07 | 7h | CSF | 2 mg/h | <0.002 | <0.002 |
| 07 | 1h | Plasma | 0.5 mg/h | <0.002 | <0.002 |
| 07 | 3h | Plasma | 0.5 mg/h | <0.002 | <0.002 |
| 07 | 5h | Plasma | 0.5 mg/h | <0.002 | <0.002 |
| 07 | 7h | Plasma | 0.5 mg/h | <0.002 | <0.002 |
| 07 | 1h | Plasma | 1 mg/h | <0.002 | <0.002 |
| 07 | 3h | Plasma | 1 mg/h | <0.002 | <0.002 |
| 07 | 5h | Plasma | 1 mg/h | <0.002 | <0.002 |
| 07 | 7h | Plasma | 1 mg/h | <0.002 | <0.002 |
| 07 | 1h | Plasma | 1.5 mg/h | <0.002 | <0.002 |
| 07 | 3h | Plasma | 1.5 mg/h | <0.002 | 0.00236 |
| 07 | 5h | Plasma | 1.5 mg/h | <0.002 | 0.00322 |
| 07 | 7h | Plasma | 1.5 mg/h | <0.002 | 0.00353 |
| 07 | 1h | Plasma | 2 mg/h | <0.002 | 0.00431 |
| 07 | 3h | Plasma | 2 mg/h | <0.002 | 0.00416 |
| 07 | 5h | Plasma | 2 mg/h | <0.002 | 0.00445 |
| 07 | 7h | Plasma | 2 mg/h | <0.002 | 0.00432 |
| 08 | 1h | CSF | 0.5 mg/h | <0.002 | 0.002 |
| 08 | 3h | CSF | 0.5 mg/h | <0.002 | 0.00234 |
| 08 | 5h | CSF | 0.5 mg/h | <0.002 | 0.00267 |
| 08 | 7h | CSF | 0.5 mg/h | <0.002 | 0.00322 |
| 08 | 1h | CSF | 1 mg/h | <0.002 | 0.00373 |
| 08 | 3h | CSF | 1 mg/h | <0.002 | 0.00406 |
| 08 | 5h | CSF | 1 mg/h | <0.002 | 0.00442 |
| 08 | 7h | CSF | 1 mg/h | <0.002 | 0.00866 |
| 08 | 1h | CSF | 1.5 mg/h | <0.002 | <0.002 |
| 08 | 3h | CSF | 1.5 mg/h | <0.002 | <0.002 |
| 08 | 5h | CSF | 1.5 mg/h | <0.002 | 0.00208 |
| 08 | 7h | CSF | 1.5 mg/h | <0.002 | <0.002 |
| 08 | 1h | CSF | 2 mg/h | <0.002 | <0.002 |
| 08 | 3h | CSF | 2 mg/h | <0.002 | <0.002 |
| 08 | 5h | CSF | 2 mg/h | <0.002 | <0.002 |
| 08 | 7h | CSF | 2 mg/h | <0.002 | <0.002 |
| 08 | 1h | Plasma | 0.5 mg/h | <0.002 | <0.002 |
| 08 | 3h | Plasma | 0.5 mg/h | <0.002 | <0.002 |
| 08 | 5h | Plasma | 0.5 mg/h | <0.002 | <0.002 |
| 08 | 7h | Plasma | 0.5 mg/h | <0.002 | <0.002 |
| 08 | 1h | Plasma | 1 mg/h | <0.002 | <0.002 |
| 08 | 3h | Plasma | 1 mg/h | <0.002 | <0.002 |
| 08 | 5h | Plasma | 1 mg/h | <0.002 | <0.002 |
| 08 | 7h | Plasma | 1 mg/h | <0.002 | <0.002 |
| 08 | 1h | Plasma | 1.5 mg/h | <0.002 | 0.00923 |
| 08 | 3h | Plasma | 1.5 mg/h | <0.002 | 0.0082 |
| 08 | 5h | Plasma | 1.5 mg/h | <0.002 | 0.00776 |
| 08 | 7h | Plasma | 1.5 mg/h | <0.002 | 0.00775 |
| 08 | 1h | Plasma | 2 mg/h | <0.002 | 0.00226 |
| 08 | 3h | Plasma | 2 mg/h | <0.002 | 0.0027 |
| 08 | 5h | Plasma | 2 mg/h | <0.002 | 0.00242 |
| 08 | 7h | Plasma | 2 mg/h | <0.002 | 0.00221 |
| 09 | 1h | CSF | 0.5 mg/h | <0.002 | <0.002 |
| 09 | 3h | CSF | 0.5 mg/h | <0.002 | <0.002 |
| 09 | 5h | CSF | 0.5 mg/h | <0.002 | <0.002 |
| 09 | 1h | CSF | 1 mg/h | <0.002 | <0.002 |
| 09 | 3h | CSF | 1 mg/h | <0.002 | <0.002 |
| 09 | 5h | CSF | 1 mg/h | <0.002 | <0.002 |
| 09 | 7h | CSF | 1 mg/h | <0.002 | <0.002 |
| 09 | 1h | CSF | 1.5 mg/h | <0.002 | <0.002 |
| 09 | 3h | CSF | 1.5 mg/h | <0.002 | <0.002 |
| 09 | 5h | CSF | 1.5 mg/h | <0.002 | <0.002 |
| 09 | 7h | CSF | 1.5 mg/h | <0.002 | <0.002 |
| 09 | 1h | CSF | 2 mg/h | <0.002 | <0.002 |
| 09 | 3h | CSF | 2 mg/h | <0.002 | <0.002 |
| 09 | 5h | CSF | 2 mg/h | <0.002 | <0.002 |
| 09 | 7h | CSF | 2 mg/h | <0.002 | <0.002 |
| 09 | 1h | Plasma | 0.5 mg/h | <0.002 | <0.002 |
| 09 | 3h | Plasma | 0.5 mg/h | <0.002 | <0.002 |
| 09 | 5h | Plasma | 0.5 mg/h | <0.002 | <0.002 |
| 09 | 7h | Plasma | 0.5 mg/h | <0.002 | <0.002 |
| 09 | 1h | Plasma | 1 mg/h | <0.002 | <0.002 |
| 09 | 3h | Plasma | 1 mg/h | <0.002 | <0.002 |
| 09 | 5h | Plasma | 1 mg/h | <0.002 | <0.002 |
| 09 | 7h | Plasma | 1 mg/h | <0.002 | <0.002 |
| 09 | 1h | Plasma | 1.5 mg/h | <0.002 | <0.002 |
| 09 | 3h | Plasma | 1.5 mg/h | <0.002 | <0.002 |
| 09 | 5h | Plasma | 1.5 mg/h | <0.002 | <0.002 |
| 09 | 7h | Plasma | 1.5 mg/h | <0.002 | <0.002 |
| 09 | 1h | Plasma | 2 mg/h | <0.002 | 0.00261 |
| 09 | 3h | Plasma | 2 mg/h | <0.002 | 0.00281 |
| 09 | 5h | Plasma | 2 mg/h | <0.002 | 0.00258 |
| 09 | 7h | Plasma | 2 mg/h | <0.002 | 0.00237 |
| 10 | 1h | CSF | 0.5 mg/h | <0.002 | <0.002 |
| 10 | 3h | CSF | 0.5 mg/h | <0.002 | <0.002 |
| 10 | 5h | CSF | 0.5 mg/h | <0.002 | <0.002 |
| 10 | 7h | CSF | 0.5 mg/h | <0.002 | <0.002 |
| 10 | 1h | CSF | 1 mg/h | <0.002 | <0.002 |
| 10 | 3h | CSF | 1 mg/h | <0.002 | <0.002 |
| 10 | 5h | CSF | 1 mg/h | <0.002 | <0.002 |
| 10 | 7h | CSF | 1 mg/h | <0.002 | <0.002 |
| 10 | 1h | CSF | 1.5 mg/h | <0.002 | <0.002 |
| 10 | 3h | CSF | 1.5 mg/h | <0.002 | <0.002 |
| 10 | 5h | CSF | 1.5 mg/h | <0.002 | <0.002 |
| 10 | 7h | CSF | 1.5 mg/h | <0.002 | <0.002 |
| 10 | 1h | CSF | 2 mg/h | <0.002 | <0.002 |
| 10 | 3h | CSF | 2 mg/h | <0.002 | <0.002 |
| 10 | 5h | CSF | 2 mg/h | <0.002 | <0.002 |
| 10 | 7h | CSF | 2 mg/h | <0.002 | <0.002 |
| 10 | 1h | Plasma | 0.5 mg/h | <0.002 | <0.002 |
| 10 | 3h | Plasma | 0.5 mg/h | <0.002 | <0.002 |
| 10 | 5h | Plasma | 0.5 mg/h | <0.002 | <0.002 |
| 10 | 1h | Plasma | 1 mg/h | <0.002 | <0.002 |
| 10 | 3h | Plasma | 1 mg/h | <0.002 | <0.002 |
| 10 | 5h | Plasma | 1 mg/h | <0.002 | <0.002 |
| 10 | 7h | Plasma | 1 mg/h | <0.002 | <0.002 |
| 10 | 1h | Plasma | 1.5 mg/h | <0.002 | <0.002 |
| 10 | 3h | Plasma | 1.5 mg/h | <0.002 | <0.002 |
| 10 | 5h | Plasma | 1.5 mg/h | <0.002 | <0.002 |
| 10 | 7h | Plasma | 1.5 mg/h | <0.002 | <0.002 |
| 10 | 1h | Plasma | 2 mg/h | <0.002 | <0.002 |
| 10 | 3h | Plasma | 2 mg/h | <0.002 | <0.002 |
| 10 | 5h | Plasma | 2 mg/h | <0.002 | <0.002 |
| 10 | 7h | Plasma | 2 mg/h | <0.002 | <0.002 |
